# Dr. Ephraim Pearson James

**Published:** 1912-05

**Authors:** 


Dr. Ephraim Pearson James of Galt, Ont., was found dead in his office, March 27, 1912. Death was supposed to be due to heart disease. He was 33 years old, a graduate of the University of Toronto, 1902. For four years, he served as ship surgeon on the Atlantic, before locating in Galt.

